# Letter to the editor: a response to Ming’s study on machine learning techniques for personalized breast cancer risk prediction

**DOI:** 10.1186/s13058-020-1255-4

**Published:** 2020-02-10

**Authors:** Daniele Giardiello, Antonis C. Antoniou, Luigi Mariani, Douglas F. Easton, Ewout W. Steyerberg

**Affiliations:** 1 Division of Molecular Pathology, The Netherlands Cancer Institute - Antoni van Leeuwenhoek Hospital, Amsterdam, The Netherlands; 2 Department of Biomedical Data Sciences, Leiden University Medical Center, Leiden, The Netherlands; 3 Department of Public Health and Primary Care, Centre for Cancer Genetic Epidemiology, University of Cambridge, Cambridge, UK; 4 Unit of Clinical Epidemiology and Trial Organization, Fondazione IRCCS Istituto Nazionale dei Tumori, Milan, Italy; 5 Department of Oncology, Centre for Cancer Genetic Epidemiology, University of Cambridge, Cambridge, UK; 6 Department of Public Health, Erasmus MC Cancer Institute, Rotterdam, The Netherlands

A recent paper [1] compared two well-known breast cancer risk prediction models (BCRAT and BOADICEA) with eight different machine learning (ML) methods. The authors found a striking improvement in cancer prediction with ML. While their comparative assessment against more classical approaches is timely, we are skeptical about the results presented.

A recent review on ML methods in a clinical epidemiological context shows that benefits of ML tend to arise in biased comparisons [2]. In the analyses of Ming et al., the ML methods were not specific for survival data and the validation process was unfair. While the ML used fits to binary outcomes prediction (having the disease or not), BOADICEA/BCRAT computes the probability of developing breast cancer over time. Regarding the second aspect, a fair comparison of the validity of the models would require data on unaffected women with prospective follow-up, with like for like risk predictions (over the same time period) for all methods. The comparisons in [1] were based on retrospective data of families of unaffected/affected individuals, and in the context of the BCRAT/BOADICEA, it is unclear what the observed and predicted events are. Furthermore, for the existing models, the study assessed external validity, while the ML methods were fitted on the same samples incorporating a tenfold cross-validation procedure, which is only equivalent to internal validation [3]. Internal validation is often overoptimistic in comparison to external validation studies [4]. Although the authors indicate the most important risk predictors in the ML approaches, the final models are not provided. A fair comparison would require the comparison of the final models from the ML with the existing models in external, prospective datasets. Moreover, discrimination is only one measure of model performance: good calibration and clinical utility assessment are also crucial [5]. Last but not least, Ming et al. did not mention which version of BOADICEA was used for the comparison with ML methods. In conclusion, the practical relevance of ML methods needs to be further investigated in this specific context, based on more rigorous methodology.

## Data Availability

Not applicable
